# Impact of Mobile Phone Usage on Sleep Quality Among Medical Students Across Latin America: Multicenter Cross-Sectional Study

**DOI:** 10.2196/60630

**Published:** 2025-02-10

**Authors:** Juan S Izquierdo-Condoy, Clara Paz, H A Nati-Castillo, Ricardo Gollini-Mihalopoulos, Telmo Raul Aveiro-Róbalo, Jhino Renson Valeriano Paucar, Sandra Erika Laura Mamami, Juan Felipe Caicedo, Valentina Loaiza-Guevara, Diana Camila Mejía, Camila Salazar-Santoliva, Melissa Villavicencio-Gomezjurado, Cougar Hall, Esteban Ortiz-Prado

**Affiliations:** 1 One Health Research Group Universidad de las Américas Quito Ecuador; 2 Grupo de Investigación Bienestar, Salud y Sociedad Escuela de Psicología y Educación Universidad de Las Américas Quito Ecuador; 3 Interinstitutional Internal Medicine Group (GIMI 1) Department of Internal Medicine Universidad Libre Cali Colombia; 4 Facultad de Medicina Universidad de Panamá Ciudad de Panamá Panama; 5 Facultad de Medicina Universidad del Pacifico Asunción Paraguay; 6 Facultad de Medicina Humana Universidad Nacional del Altiplano Puno Peru; 7 Facultad de Ciencias de la Salud Universidad Privada Franz Tamayo La Paz Bolivia; 8 Facultad de Ciencias de la Salud Universidad del Quindío Armenia Colombia; 9 Facultad de Medicina Institución Universitaria Visión de las Américas Pereira Colombia; 10 Public Health Department Bringham Young University Provo, UT United States

**Keywords:** mobile phone, addiction behavior, sleep quality, medical students, Latin America

## Abstract

**Background:**

The ubiquitous use of mobile phones among medical students has been linked to potential health consequences, including poor sleep quality.

**Objective:**

This study investigates the prevalence of mobile phone addiction and its association with sleep quality among medical students across 6 Latin American countries.

**Methods:**

A descriptive, cross-sectional, multicenter study was conducted between December 2023 and March 2024 using a self-administered online survey. The survey incorporated the Mobile Phone Addiction Scale and the Pittsburgh Sleep Quality Index to evaluate mobile phone addiction and sleep quality among 1677 medical students. A multiple regression model was applied to analyze the relationship between mobile phone addiction and poor sleep quality, adjusting for sex, age, and educational level to ensure robust results.

**Results:**

Mobile phone addiction was identified in 32.5% (545/1677) of participants, with significant differences across countries. The overall mean Pittsburgh Sleep Quality Index score was 7.26, indicating poor sleep quality. Multiple regression analysis revealed a strong association between mobile phone addiction and poor sleep, controlled for demographic variables (β=1.4, 95% CI 1.05-1.74).

**Conclusions:**

This study underscores a significant prevalence of mobile phone addiction among medical students and its detrimental association with sleep quality in Latin America. The findings advocate for the need to address mobile phone usage to mitigate its negative implications on student health and academic performance. Strategies to enhance digital literacy and promote healthier usage habits could benefit medical education and student well-being.

## Introduction

By 2024, approximately 17.8 billion mobile phones are projected to be in use globally, according to Statista [1]. Initially designed for voice communication, mobile phones have evolved into essential tools for text messaging, complex communication, and daily activities [2]. The advent of smartphones with advanced computational capabilities has further integrated these devices into modern life [3]. While they offer substantial benefits, such as enhanced access to educational and informational resources, excessive use also poses significant risks [4]. Acute or chronic overuse of mobile phones has been associated with physical issues like headaches, carpal tunnel syndrome, tendinitis, and accidents caused by distraction [5]. In addition, excessive use can result in less overt but impactful effects, including reduced motivation, impaired memory and concentration, sleep disturbances, and learning difficulties [6].

One of the most well-documented negative consequences of mobile phone usage is its disruption of sleep patterns and quality. Prolonged screen exposure and late-night cellphone use adversely affect both the quantity and quality of sleep [7,8]. Poor sleep has been linked to a range of health problems, including cardiovascular diseases, mental health disorders, neurodegenerative conditions, musculoskeletal issues, and a reduced overall quality of life [9,10]. University students, particularly those belonging to Generation Z (Gen Z), also known as iGen or postmillennials, face compounded risks. Born between 1996 and 2015, Gen Z is the first generation to grow up with the internet and portable digital technology. These “digital natives” excel in mobile phone use and social media interaction but have less experience with traditional communication methods, such as print media. For medical students, the demands of their academic schedules, combined with excessive mobile phone usage, pose risks to mental health, sleep quality, and academic performance [11].

In recent years, mobile phones have become indispensable tools in the academic training of medical students [12]. Originally designed for personal communication, these devices have evolved into essential academic resources, facilitating learning and collaboration. However, this transition has also been associated with dependency and excessive use, raising concerns about potential addiction [13]. Constant internet connectivity provided by smartphones fosters engagement not only with educational resources but also with addictive behaviors and content [3]. These include compulsive information seeking, excessive use of online games and shopping platforms, and problematic online interactions such as cybersexuality and cybercontacts [14]. For medical students, in particular, problematic smartphone use, nomophobia (a fear of not having mobile phone connectivity), and general dependency not only negatively impacts academic performance but also poses significant health risks [15-18].

Recent studies in the United States and Turkey have identified a correlation between increased mobile phone use and poor sleep quality among university and medical students [19,20]. A meta-analysis further highlighted that sleep quality is closely linked to academic performance in medical students, emphasizing the need for interventions to address this issue [14]. Despite this growing body of evidence, research on the impact of mobile phone addiction on sleep quality among medical students in Latin America remains limited.

Medical students in Latin America face unique challenges, including demanding schedules, irregular sleep patterns, and high stress levels, which may make them particularly vulnerable to mobile phone addiction. In addition, the reliance on mobile phones for communication and education in resource-limited health care education systems could exacerbate these issues.

This study aims to investigate the prevalence of mobile phone addiction and its association with sleep quality among medical students in 6 Latin American countries. By addressing this gap, we hope to provide insights that can inform targeted interventions to improve the well-being and academic success of medical students in the region.

## Methods

### Study Design

A descriptive, cross-sectional, multicenter study was conducted from December 2023 to March 2024.

### Setting and Participants

This study used a self-administered online survey targeting medical students enrolled at universities across 6 Latin American countries: Bolivia, Colombia, Ecuador, Panama, Paraguay, and Peru. Participants included undergraduate students from the first to the final years (sixth or seventh) of their medical degree. Participants were selected using a nonprobability convenience sampling method through the online survey platform SurveyMonkey. Participation was voluntary.

### Survey Development and Measures

The research team designed a 49-item anonymous online survey to collect data on demographic characteristics, mobile phone usage, and sleep quality among the Latin American medical student population. An initial version of the questionnaire was reviewed by a public health expert to ensure its relevance, accuracy, and to preempt potential errors. It was then pilot tested with 20 medical students from Ecuador to fine tune comprehension and design; data from this phase were excluded from the final analysis. Revisions after pilot test produced the final version of the survey, which was drafted in Spanish. The report on the online survey used in this research, prepared following the CHERRIES (Checklist for Reporting Results of Internet E-Surveys), is provided in Table S1 in Multimedia Appendix 1.

### Questionnaire and Variables

The final version of the questionnaire was structured into 3 sections to meet the research objectives.

#### Demographic Data

The first section collected demographic information such as sex, age, country of residence, year of study, and type of university, categorized by funding source (public or private). It also included an additional year category (7th year) to account for students in countries such as Colombia and Panama, where medical training can extend up to 13 semesters.

#### Mobile Phone Addiction

The second section used the Mobile Phone Addiction Scale, designed and validated by Basu et al [21], which adheres to *ICD-10* (*International Statistical Classification of Diseases, Tenth Revision*) criteria for substance dependence syndrome. It comprised 20 questions rated on a 6-point Likert scale (from 1-completely disagree to 6-completely agree). The questionnaire evaluated 6 addiction components: intense desire, poor control, abstinence, tolerance, diminished interest in alternative pleasures, and harmful use. Addiction presence was defined if half or more of the questions in each component were affirmed; global addiction was noted when three or more components were present.

#### Sleep Quality

The third section used the standardized Pittsburgh Sleep Quality Index (PSQI) [22,23], designed to evaluate sleep quality through 19 questions covering 7 components: subjective sleep quality, sleep latency, sleep duration, habitual sleep efficiency, sleep disturbances, use of sleep medication, and daytime dysfunction. Scores ranged from 0 (no difficulty) to 3 (very severe difficulty), with a total score from 0 to 21. A score of 5 or less indicated normal rest, while scores greater than 5 indicated poor rest, categorizing the overall sleep quality level.

### Data Collection and Management

**Figure 1:**
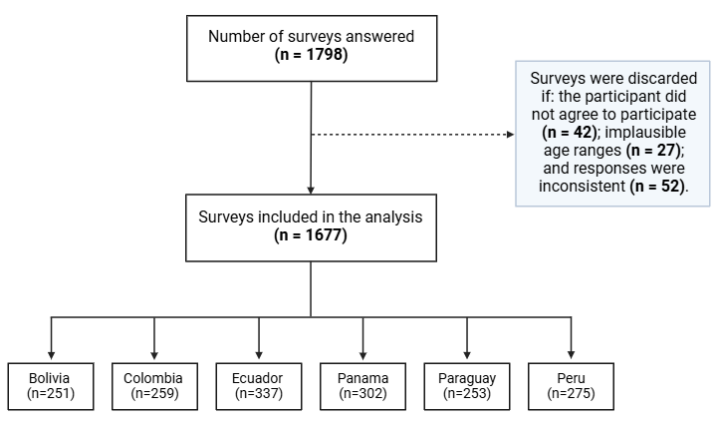
Flowchart of the Strengthening the Reporting of Observational Studies in Epidemiology–based sample selection process for this study.

The online platform SurveyMonkey was used to facilitate data collection. Participants accessed the survey through a unique link shared across social media channels, including Facebook and WhatsApp (Meta). The preamble of the survey outlined the study’s aims, assured confidentiality, and sought informed consent. Participants were required to agree to the informed consent and accept the participation agreement to proceed, reinforcing the survey’s anonymity. To ensure high data integrity, all responses were meticulously reviewed for potential errors or inconsistencies, such as implausible age ranges or respondents selecting all available answers. This scrutiny led to the exclusion of questionnaires from an original sample of 1798 survey responses before filtering to a final total of 1677 responses which were considered valid and included in the study (Figure 1).

### Bias

To mitigate potential bias during data collection and management, several strategies were used. For example, IP addresses were used to eliminate duplicate submissions by configuring the SurveyMonkey platform to limit 1 response per IP address. To ensure anonymity the questionnaire was carefully curated to avoid collecting any identifiable information, including IP addresses, beyond what was necessary for filtering duplicates. In the analysis phase, additional steps were taken to minimize bias. One of the researchers meticulously reviewed the results, and any discrepancies identified were collaboratively resolved. This rigorous approach ensured that only valid and genuine responses were included in the final analysis, substantially enhancing the reliability and credibility of the study’s findings. The use of a nonprobability convenience sampling method was justified in this study of medical students across 6 Latin American countries due to logistical, resource, and time constraints. This method enabled the inclusion of participants from multiple universities, making the study feasible and cost-effective while capturing valuable insights into issues such as mobile phone addiction and sleep quality. Although convenience sampling introduces potential biases, including selection bias and limited generalizability, it was the most practical option given the difficulty of accessing a more representative sample across diverse regions. Despite these limitations, the approach allowed for timely data collection and provided exploratory insights into the specific challenges faced by medical students.

### Statistical Analysis

The current study used descriptive statistical tests to analyze the responses for each categorical variable, including the calculation of frequencies and percentages. For quantitative variables, measures of central tendency (mean) and dispersion (SD) were evaluated.

To evaluate the relationships between the variables studied, inferential statistical tests were used. Chi-square tests were conducted to assess the influence of participant characteristics on the presence of mobile phone addiction. *t* tests and one-way ANOVA were used to examine the influence of characteristics on sleep quality as determined by mean PSQI scores.

Ordinary least squares (OLS) regression was conducted as part of a multiple regression model adjusted for sex, age, and educational level was used to estimate the association between mobile phone addiction and poor sleep quality, expressed by β and 95% CI. In all statistical analyses, a *P* value of <.05 was considered statistically significant. All the analysis was conducted using R (R Foundation for Statistical Computing).

### Ethical Considerations

This study was conducted in strict adherence to the ethical standards set forth in the Declaration of Helsinki. It also followed the ethical protocols approved by the Ethics Committee of Universidad de Las Américas, under code 2023-EXC-004. The research team was trained to ensure that all aspects of the study upheld the principles of participant anonymity and voluntary participation. No personally identifiable or sensitive information was solicited or included in the research data.

## Results

### Demographic Characteristics

A total of 1677 medical students from 6 Latin American countries participated in this study. Ecuador and Panama had the highest representations at 20.1% and 18.0%, respectively. The majority of participants were female (1083/1677, 64.4%), and over half (973/1677, 58%) were in their initial years of undergraduate study (1st to 3rd year). The majority (967/1677, 57.7%) were enrolled in private universities (Table 1).

**Table 1:** Demographic characteristics of medical students from 6 Latin American countries.

Characteristics			Bolivia (n=251		Colombia (n=259)		Ecuador (n=337)		Panama (n=302)		Paraguay (n=253)		Peru (n=275)		Overall (n=1677)
**Sex, n (%)**															
	Male	99 (39.4)		103 (39.8)		118 (35)		113 (37.4)		73 (28.9)		88 (32)		594 (35.4)	
	Female	152 (60.6)		156 (60.2)		219 (65)		189 (62.6)		180 (71.1)		187 (68)		1083 (64.6)	
**Age, mean (SD)**															
		20.2 (3.14)		21.6 (3.46)		21.7 (2.18)		22.1 (2.27)		23 (4.58)		22.3 (3.76)		21.8 (3.36)	
**Year of study, n (%)**															
	First	111 (44.2)		31 (12)		12 (3.6)		22 (7.3)		67 (26.5)		35 (12.7)		278 (16.6)	
	Second	104 (41.4)		51 (19.7)		43 (12.8)		52 (17.2)		33 (13.0)		64 (23.3)		347 (20.7)	
	Third	28 (11.2)		53 (20.5)		124 (36.8)		60 (19.9)		35 (13.8)		48 (17.5)		348 (20.8)	
	Fourth	4 (1.6)		34 (13.1)		124 (36.8)		20 (6.6)		58 (22.9)		38 (13.8)		278 (16.6)	
	Fifth	2 (0.8)		65 (25.1)		14 (4.2)		41 (13.6)		32 (12.6)		33 (12)		187 (11.2)	
	Sixth	2 (0.8)		18 (6.9)		8 (2.4)		102 (33.8)		20 (7.9)		19 (6.9)		169 (10.1)	
	Seventh	0 (0)		7 (2.7)		12 (3.6)		5 (1.7)		8 (3.2)		38 (13.8)		70 (4.2)	
**Level of education, n (%)**															
	Initial (first-third year)	243 (96.8)		135 (52.1)		179 (53.1)		134 (44.4)		135 (53.4)		147 (53.5)		973 (58)	
	Advanced (fourth-seventh year)	8 (3.2)		124 (47.9)		158 (46.9)		168 (55.6)		118 (46.6)		128 (46.5)		704 (42)	
**Type of university, n (%)**															
	Private university	248 (98.8)		89 (34.4)		256 (76%)		41 (13.6%)		229 (90.5)		104 (37.8)		967 (57.7)	
	Public university	3 (1.2)		170 (65.6)		81 (24%)		261 (86.4%)		24 (9.5)		171 (62.2)		710 (42.3)	

### Smartphone Usage

**Figure 2:**
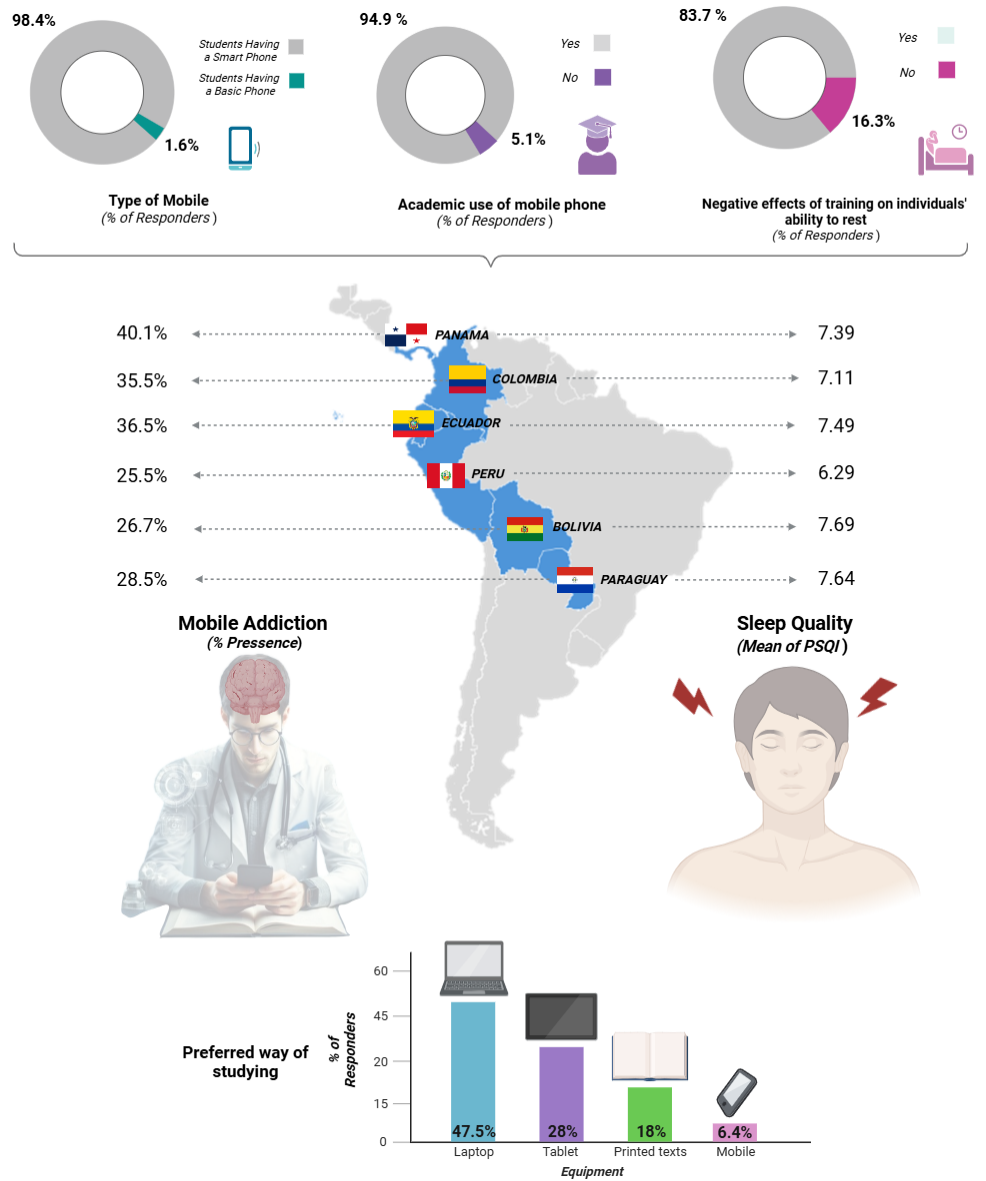
Characteristics of cell phone use, academic preparation, and distribution of cell phone addiction and sleep quality by country of residence among Latin American medical students.

Smartphones were used by nearly the entire study population (1651/1677, 98.4%), with 94.9% (1591/1677) reporting using mobile phones for academic purposes. However, computers or laptops were preferred for studies (797/1677, 47.5%), while mobile phones were the least preferred option for studying (108/1677, 6.4%; Figure 2).

### Mobile Phone Addiction

According to the Mobile Phone Addiction Scale, 32.5% (545/1677) of participants exhibited mobile phone addiction. The domain most affected was tolerance to use (974/1677, 58.1%), followed by an intense desire to use the phone (708/1677, 42.2%). A decrease in pleasure was the least common symptom, noted in only 9.8% (165/1677) of the sample (Table S2 in Multimedia Appendix 1).

According to the distribution among the participating countries, the highest prevalence of mobile phone addiction was observed in medical students from Panama (121/302, 40.1%), while the lowest was in Peru (70/253, 25.5%; *P*<.001; Figure 2). Furthermore, the presence of mobile phone addiction was associated with certain participant characteristics, being significantly higher (38/86, 44.2%) among medical students who stated they did not use their phones for academic purposes (*P*=.02), as well as among those who reported that their undergraduate preparation negatively affected their rest (448/1251, 35.8%; *P*<.001). In addition, medical students who preferred printed texts had the lowest percentage of mobile phone addiction (81/302, 22.8%) when compared with those participants using screen devices (mobile phone, computer, and tablet; *P*=.002; Table 2).

**Table 2:** Relationship between the characteristics of the participants with mobile phone addiction and sleep quality.

Characteristics		Mobile addiction				PSQI^a^	
		Absence, n (%)	Presence, n (%)	*P* value	Mean (SD)		*P* value
**Sex**				.92			.02
	Male (n=594)	400 (67.3)	194 (32.7)		7.04 (3.34)		
	Female (n=1083)	732 (67.6)	351 (32.4)		7.39 (3.5)		
**Level of education**				.85			.22
	Initial (n=973)	655 (67.3)	318 (32.7)		7.37 (3.41)		
	Advanced (n=704)	477 (67.8)	227 (32.2)		7.11 (3.5)		
**Type of university**				.58			.001
	Private university (n=967)	658 (68)	309 (32)		7.57 (3.48)		
	Public university (n=710)	474 (66.8)	236 (33.2)		6.85 (3.37)		
**Academic mobile phone use**				.02			.72
	No (n=86)	48 (55.8)	38 (44.2)		7.40 (3.5)		
	Yes (n=1591)	1084 (68.1)	507 (31.9)		7.26 (3.45)		
**Preferred way of studying**				.002			.02
	Mobile phone (n=108)	61 (56.5)	47 (43.5)		8.21 (3.71)		
	Computer/laptop (n=797)	551 (69.1)	246 (30.9)		7.13 (3.41)		
	Tablet/iPad (n=470)	299 (63.6)	171 (36.4)		7.22 (3.47)		
	Prefer printed texts (n=302)	221 (73.2)	81 (22.8)		7.34 (3.4)		
**Negative effects of your academic training on your rest**				<.001			<.001
	No (n=426)	329 (77.2)	97 (22.8)		6.08 (3.63)		
	Yes (1251)	803 (64.2)	448 (35.8)		7.49 (3.37)		

^a^PSQI: Pittsburgh Sleep Quality Index.

### Sleep Quality

As measured by the PSQI, a general mean of poor sleep quality of 7.26 (SD 3.45) was found among medical students from the 6 study countries. The components with the highest scores (indicating the worst sleep quality) were subjective sleep quality (1.33, SD 0.77) and daytime dysfunction (1.31, SD 0.84), while the lowest scores were found in the use of sleep medication (0.27, SD 0.70) and habitual sleep efficiency (0.89, SD 1.12; Table 3).

**Table 3:** Characteristics of the components of sleep quality in medical students in Latin America.

PSQI^a^ values		Bolivia (n=251), mean (SD)	Colombia (n=259), mean (SD)	Ecuador (n=337), mean (SD)	Panama (n=302), mean (SD)	Paraguay (n=253), mean (SD)	Peru (n=275), mean (SD)	Overall (n=1677), mean (SD)
PSQI		7.69 (3.61)	7.11 (3.43)	7.47 (3.31)	7.39 (3.49)	7.64 (3.52)	6.29 (3.22)	7.26 (3.45)
**Sleep quality components**								
	Subjective sleep quality	1.40 (0.87)	1.31 (0.75)	1.31 (0.69)	1.42 (0.74)	1.38 (0.8)	1.13 (0.75)	1.33 (0.77)
	Sleep latency	1.15 (0.91)	1.20 (0.97)	1.11 (0.86)	1.08 (0.97)	1.39 (0.97)	0.996 (0.88)	1.15 (0.93)
	Sleep duration	1.33 (1.13)	1.09 (1.05)	1.23 (1.04)	1.23 (1.09)	1.01 (1.07)	0.924 (1.01)	1.14 (1.07)
	Habitual sleep efficiency	0.95 (1.16)	0.76 (1.05)	0.88 (1.09)	0.96 (1.16)	0.97 (1.15)	0.79 (1.07)	0.89 (1.12)
	Sleep disturbance	1.31 (0.66)	1.18 (0.54)	1.26 (0.57)	1.04 (0.5)	1.21 (0.49)	1.16 (0.55)	1.19 (0.56)
	Use of sleeping medication	0.25 (0.6)	0.29 (0.73)	0.27 (0.72)	0.25 (0.68)	0.38 (0.86)	0.17 (0.55)	0.27 (0.7)
	Daytime dysfunction	1.29 (0.9)	1.28 (0.78)	1.41 (0.84)	1.43 (0.86)	1.28 (0.79)	1.11 (0.81)	1.31 (0.84)

^a^PSQI: Pittsburgh Sleep Quality Index.

Differences in sleep quality by country showed the highest scores for Bolivian (7.69, SD 3.61) and Paraguayan (7.64, SD 3.52) students, while Peru had the lowest score (6.29, SD 3.22; *P*<.001). Complementary post hoc analyses revealed that Peruvian medical students had significantly lower PSQI scores (better sleep quality) compared with their counterparts from Bolivia (*P*<.001), Ecuador (*P*<.001), Panama (*P*=.001), and Paraguay (*P*<.001; Figure 2).

Furthermore, characteristics associated with higher PSQI scores (worse sleep quality) were found in female medical students (7.39, SD 3.5; *P*=.02), those from private universities (7.57, SD 3.48; *P*=.001), those who preferentially use mobile phones for studying (8.21, 3.71; *P*=.02), and those who consider that academic preparation affects their sleep quality (7.49, SD 3.37; *P*<.001; Table 2).

### Effect of Mobile Phone Addiction on Sleep Quality

The negative influence of mobile phone addiction on sleep quality was statistically significant across all seven components of the PSQI, showing higher mean scores (worse quality) in the group of medical students with mobile phone addiction, including in the dimensions of habitual sleep efficiency and use of sleeping medication (Table 4).

**Table 4:** Effect of mobile phone addiction on the components of sleep quality of medical students in Latin America.

Components	Mobile addiction		*P* value
	Absence (n=1132), mean (SD)	Presence (n=545), mean (SD)	
Subjective sleep quality	1.27 (0.76)	1.44 (0.76)	<.001
Sleep latency	1.08 (0.9)	1.28 (0.97)	<.001
Sleep duration	1.06 (1.05)	1.29 (1.09)	<.001
Habitual sleep efficiency	0.83 (1.09)	1 (1.17)	.003
Sleep disturbance	1.15 (0.54)	1.29 (0.59)	<.001
Use of sleeping medication	0.23 (0.67)	0.33 (0.77)	.01
Daytime dysfunction	1.19 (0.81)	1.56 (0.84)	<.001

A multiple regression was testes to demonstrated that mobile phone addiction is a factor associated with poor sleep quality as measured by mean PSQI scores even after adjusting for sex, age, and level of studies. The model largely met the assumptions of OLS regression, including linearity, independence, and homoscedasticity. However, the normality test of the residuals did not fully satisfy the assumption of normality. Despite this, we chose to retain the OLS model for several reasons. First, OLS is robust to deviations from normality, particularly when the sample size is large, as was the case in this study. Second, the central limit theorem suggested that the distribution of the residuals would approximate normality with a sufficiently large sample, even if the data were not perfectly normally distributed. Finally, the main goal of this model was to estimate the relationships between variables, and OLS provided unbiased, consistent, and efficient estimates under the assumption of homoscedasticity and linearity, which the model satisfied. Therefore, despite the minor concern regarding normality, the OLS model remained appropriate for the analysis. The model demonstrated the relationship between mobile phone usage and sleep quality among medical students in the study (β=1.4, 95% CI 1.05-1.74; Table 5).

**Table 5:** Regression analysis of mobile phone addiction impact on sleep disturbance (by Pittsburgh Sleep Quality Index mean scores). Analyses of sleep disturbances predicted by cell phone were controlled for gender, age and educational level (R2=0.06).

Variables		Beta values (95% CI)
Mobile phone addiction		1.40 (1.05 to 1.74)^a^
Sex (female)		0.40 (0.06 to 0.74)^b^
Age		0.32 (0.13 to 0.51)^a^
Level of education (advanced)		–0.46 (–0.85 to –0.06)^b^
**Country**		
	Colombia	–0.62 (–1.23 to –0.02)^b^
	Ecuador	–0.31 (–0.88 to –0.26)
	Panama	–0.42 (–1.02 to –0.17)
	Paraguay	–0.17 (–0.79 to –0.44)
	Peru	–1.41 (–2 to –0.81)^a^

^a^*P*<.001.

^b^*P*<.05.

## Discussion

### Principal Findings

By the third decade of the 21st century, access to and use of mobile phones has become nearly universal, a reality that includes medical students in Latin America [24]. This research aimed to explore mobile phone addiction among medical students across 6 Latin American countries, as well as to evaluate their sleep quality and the potential impact of excessive and unhealthy mobile phone use on sleep.

This multicenter study included 1677 undergraduate medical students, revealing a notable predominance of female participants. This finding aligns with recent research indicating that a majority of medical students in Latin America are female [25,26]. Nearly 100% of participants reported using a smartphone, a rate comparable with those reported among medical students in other regions [27]. In addition, 94.9% of participants used their devices for academic purposes, a figure higher than in other regions but consistent with global trends favoring the academic use of mobile phones among undergraduate medical students [12,28,29].

Although smartphones are primarily associated with barrier-free communication through social networks, they also possess numerous tools that are increasingly integrated into academic training, particularly among medical students. However, prolonged and uncontrolled use of these devices can have significant health effects on users [30-32]. Medical students are uniquely affected by mobile phone addiction and poor sleep quality due to the combination of demanding academic schedules, high stress levels, and the pervasive nature of digital technology. The rigorous nature of medical training often necessitates extensive study hours, leading students to rely heavily on smartphones for quick access to educational materials. This reliance, coupled with the need for social interaction and recreation through the same devices, often results in prolonged and uncontrolled usage.

Despite being regarded as a tool for academic enhancement, approximately one-third (32.5%) of the study population exhibited mobile phone addiction, with rates ranging from 25.5% among Peruvian students to 40.1% among Panamanian students. Comparing addiction rates globally is challenging due to variations in the questionnaires used to assess mobile phone use, addiction, or overuse, such as the Smartphone Addiction Scale (SAS-SV) or the Cell Phone Overuse Scale (COS) [33]. For example, Kamal et al [34] reported a 48% addiction rate among Pakistani medical and dental students using a similar instrument, while other studies found addiction rates ranging from 10.7% among Iranian medical students [35] to 34.4% among first and second-year medical students in Srinagar, India [36]. The questionnaire used in the present study [21] found that addiction was most commonly characterized by tolerance (using the phone more than expected and considering the usage excessive) and a strong desire to use the phone, particularly for immediate interaction on social networks or phone use even when others are nearby, traits typical of addictive behaviors [37]. Despite the heterogeneity in assessment tools, the body of research underscores the pervasive nature of mobile phone addiction among medical students worldwide, including in Latin America.

Interestingly, this study found no significant demographic variables related to overuse or addiction to mobile phones, such as gender [38,39]. However, there was a concerning relationship between students who did not use mobile phones for academic purposes and higher rates of addiction, potentially due to a preference for leisure activities such as social networking, gaming, or streaming platforms [40]. In addition, students who reported that their studies negatively affected their rest exhibited higher rates of addiction, suggesting an indirect relationship between mobile phone addiction and poor sleep. This notion is supported by findings showing that students who preferred printed texts for studying had lower addiction rates.

Sleep quality studies have become more standardized with the widespread acceptance of the PSQI. The average PSQI score among students in this study (7.26, SD 3.45) indicated poor sleep quality, considerably higher than scores reported in similar studies, including military medical students (PSQI score=5.78, SD 2.26) [41], Indian medical students (PSQI score=4.43, SD 2.62) [39], and Iranian medical students (PSQI score=5.38, SD 2.31). Systematic reviews report meta-mean PSQI scores between 5.95 and 6.1 [33,42]. Poor sleep quality among medical students is well-documented and has been attributed to heavy study loads, irregular schedules, and a preference for study over sleep [43,44]. In this study, students from private universities and women exhibited worse sleep quality, a trend previously observed in other research [41].

There was a significant and consistent association between mobile phone addiction and poor sleep quality, with addicted students scoring higher across all PSQI dimensions (subjective sleep quality, sleep latency, sleep duration, habitual sleep efficiency, sleep disturbances, use of sleep medication, and daytime dysfunction) compared with those without addiction (*P*<.05). Regression analyses revealed a global association between mobile phone addiction and diminished sleep quality, even after adjusting for sex, age, and educational level (β=1.4; 95% CI 1.05-1.74; *P*<.001). This association has been widely evidenced in previous studies of medical students across different countries and academic levels [33,35,38,39,41,45].

The findings of this study offer several plausible explanations for the observed association between mobile phone addiction and poor sleep quality. Smartphones facilitate a wide range of activities, including gaming, streaming, and social media use, all of which compete for users’ limited time and attention, often displacing sleep. Social media platforms, in particular, are deliberately designed to capture and retain user engagement through features such as “infinite scroll,” notifications, direct messaging (DMs), and interaction metrics like likes, follows, and shares [46,47]. These features create an attention-driven ecosystem where users are incentivized to remain active, with their engagement monetized for advertising purposes [46].

In addition, the blue light emitted by smartphones has been widely recognized as a disruptor of circadian rhythms [48-50]. Artificial light exposure, particularly at night, delays the release of melatonin, the hormone responsible for regulating sleep-wake cycles, exacerbating sleep disturbances. Among medical students, this effect is particularly concerning due to their already irregular schedules and high stress levels [51-53]. The combination of attention-capturing smartphone apps and blue light exposure likely contributes to prioritizing phone use oversleep, providing a compelling explanation for the current study’s findings.

This study highlights a critical issue, emphasizing the dual impact of mobile phones on the present and future of academic medical training [54]. While these devices provide significant benefits, including portable and digital access to textbooks, scientific articles, and practical tools such as video content, infographics, and podcasts [24,55-57], heir use also presents risks. These resources can enhance knowledge acquisition, even in resource-limited settings like Latin America. However, decision-makers, including university institutions and educators, must be cognizant of the potential negative consequences of mobile phone use in academic environments. These include cross-device addiction, indirect effects on sleep quality, and other physical and mental health challenges.

The broader implications of these findings extend beyond sleep disturbances. Poor sleep quality and mobile phone addiction can significantly impair cognitive function, emotional regulation, and physical health, which are vital for medical students' academic success and future professional practice. Sleep deprivation and distractions caused by addiction may hinder students' ability to learn effectively, retain critical information, and perform competently in clinical settings, ultimately impacting patient care.

Addressing these challenges requires urgent intervention. Institutions should integrate sleep education into medical curricula, emphasizing its importance for cognitive performance and overall well-being. Behavioral strategies, such as implementing “digital detox” periods or encouraging the use of apps that monitor and limit screen time, can help students manage their smartphone use. Furthermore, counseling and support services tailored to stress management and time optimization can address the underlying causes of overuse and improve sleep hygiene.

### Limitations

This study has several limitations that should be acknowledged. First, the survey was conducted online, which may have introduced a sampling bias, favoring medical students with easier access to technology or greater familiarity with online platforms. In addition, the reliance on self-reported data poses the risk of social desirability bias, where participants may exaggerate their academic use of smartphones or provide responses they perceive as socially acceptable.

The subjective sleep assessments used in this study, specifically the PSQI, are practical, cost-effective, and widely used in population-based research due to their ease of administration and scalability. However, subjective measures are inherently limited by recall bias and participants’ ability to accurately evaluate their sleep habits. In contrast, objective sleep assessment methods, such as actigraphy and polysomnography, provide direct and measurable data on sleep parameters and are considered more precise. These objective tools often reveal discrepancies compared with subjective assessments, as personal perceptions may lead to overestimation or underestimation of sleep duration or quality. Nevertheless, objective methods may fail to account for psychological factors influencing perceived sleep quality, which subjective tools can capture.

In this study, the reliance solely on subjective measures, such as the PSQI, represents a limitation. This approach may not fully reflect the complexity of sleep patterns or detect potential clinical sleep disturbances. Future research should consider integrating objective assessments to complement and validate self-reported sleep data, providing a more comprehensive understanding of sleep behaviors. Furthermore, the cross-sectional design of this study precludes the establishment of causality between mobile phone addiction and poor sleep quality. It remains unclear whether academic mobile phone use contributes to addiction and reduced sleep quality, or if academically successful students inherently use smartphones more frequently and experience shorter sleep durations.

Finally, while the study included participants from 6 Latin American countries, the findings may not be universally generalizable across the entire region due to cultural, economic, and educational differences. Future studies should aim to include more diverse populations to enhance the external validity of the results.

### Conclusions

In conclusion, this study reveals a substantial prevalence of mobile phone addiction among medical students from 6 Latin American countries, affecting approximately one-third of the sample. In addition, the overall sleep quality of student participants was found to be poor, as indicated by a mean PSQI score higher than previously reported. Findings suggest that using mobile phones for studying can indirectly contribute to poorer sleep quality by diverting attention to other activities during study sessions. This underscores the dual nature of mobile technology, which can serve both educational and recreational purposes, each with differing impacts on the lives and health of students. Importantly, we identified a significant association between mobile phone addiction and poor sleep quality. Overall, this study confirms the widespread academic integration of mobile phones among medical students in Latin America and highlights the significant need for strategies to address the use of these devices in academia and their potential impact on health and academic success.

## Appendix

Multimedia Appendix 1 Checklist for Reporting Results of Internet E-Surveys (CHERRIES).

## Abbreviations

CHERRIES: Checklist for Reporting Results of Internet E-Surveys
COS: Cell Phone Overuse Scale
DM: direct message
Gen Z: generation Z
ICD-10: International Statistical Classification of Diseases, Tenth Revision
OLS: ordinary least squares
PSQI: Pittsburgh Sleep Quality Index
SAS-SV: Smartphone Addiction Scale-Short Version
