# Supplementary material for: Impact of Mobile Phone Usage on Sleep Quality Among Medical Students Across Latin America: Multicenter Cross-Sectional Study
Source: J Med Internet Res. 2025 Feb 10;27:e60630. doi: 10.2196/60630 (PMC11851046; doi:10.2196/60630)
Supplement: Multimedia Appendix 1 [file jmir_v27i1e60630_app1.docx]

**Supplementary Table 1.** Checklist for Reporting Results of Internet E-Surveys (CHERRIES) for our study.

| **Item Category** | **Checklist Item** | **Explanation** |
| --- | --- | --- |
| **Design** | | |
|  | Describe survey design | This study employed a descriptive, cross-sectional, multicenter design targeting medical students from six Latin American countries (Bolivia, Colombia, Ecuador, Panama, Paraguay, and Peru). A non-probability convenience sampling method was used |
| **IRB (Institutional Review Board) approval and informed consent process** | | |
|  | IRB approval | The study received ethical approval from the Ethics Committee of Universidad de Las Américas (CEISH-UDLA) under code 2023-EXC-004. |
|  | Informed consent | Participants were informed about the study's purpose, expected completion time, and data confidentiality through a preamble in the online survey. Participation was voluntary, and informed consent was obtained before survey access by requiring agreement to the Participation Agreement. |
|  | Data protection | Anonymity was maintained by avoiding the collection of personally identifiable information, including IP addresses beyond their use to prevent duplicates. Data were stored securely on the SurveyMonkey platform and accessible only to the research team. |
| **Development and pre-testing** | | |
|  | Development and testing | The survey was developed by the research team and consisted of a 49-item questionnaire. It was reviewed by a public health expert and pilot tested with 20 medical students from Ecuador for usability and comprehension. Feedback from the pilot test informed revisions before the final deployment. |
| **Recruitment process and description of the sample having access to the questionnaire** | | |
|  | Open survey versus closed survey | The survey was open, with links shared publicly through social media platforms. |
|  | Contact mode | Recruitment occurred online via unique survey links disseminated through Facebook and WhatsApp. |
|  | Advertising the survey | Recruitment messages specified eligibility criteria, study purpose, and voluntary participation. These messages were shared across social media groups and personal networks. |
| **Survey administration** | | |
|  | Web/E-mail | The survey was administered via the online platform SurveyMonkey. |
|  | Context | Recruitment links were shared broadly across social media platforms, ensuring access to a diverse pool of medical students across multiple countries. |
|  | Mandatory/voluntary | Participation was entirely voluntary. |
|  | Incentives | No incentives were offered. |
|  | Time/Date | Data collection was conducted from December 2023 to March 2024. |
|  | Randomization of items or questionnaires | No randomization was applied. |
|  | Adaptive questioning | Adaptive questioning was not used. |
|  | Number of Items | The survey contained 49 items. |
|  | Number of screens (pages) | The questionnaire was divided into three sections corresponding to demographic information, mobile phone addiction, and sleep quality. |
|  | Completeness check | Mandatory fields ensured critical data completeness. Responses were checked for internal consistency during data review. |
|  | Review step | Participants could review and revise their answers before submission using the "Back" button. |
| **Response rates** | | |
|  | Unique site visitor | Unique visitors were tracked based on IP addresses. |
|  | View rate (Ratio of unique survey visitors/unique site visitors) | Not Applicable. |
|  | Participation rate (Ratio of unique visitors who agreed to participate/unique first survey page visitors) | 1,798 participants initially started the survey, and 1,667 valid responses were included, indicating a participation rate of approximately 92.7%. |
|  | Completion rate (Ratio of users who finished the survey/users who agreed to participate) | The completion rate was approximately 92.7% (1,667 completed responses from 1,798 initial submissions). |
| **Preventing multiple entries from the same individual** | | |
|  | Cookies used | Cookies were not used for tracking participants. |
|  | IP check | The platform restricted multiple entries from the same IP address to prevent duplicate responses. |
|  | Log file analysis | No additional log file analysis was conducted. |
|  | Registration | Not applicable, as this was an open survey. |
| **Analysis** | | |
|  | Handling of incomplete questionnaires | Only fully completed questionnaires were analyzed. Questionnaires with incomplete or inconsistent responses were excluded. |
|  | Questionnaires submitted with an atypical timestamp | Responses submitted in implausibly short times were flagged and excluded. |
|  | Statistical correction | No statistical weighting or propensity score adjustments were applied. Descriptive and inferential statistical analyses were conducted using R software (2020 version). |
